# Clinical characteristics and prognostic factors analysis of core binding factor acute myeloid leukemia in real world

**DOI:** 10.1002/cam4.6693

**Published:** 2023-12-07

**Authors:** Yamei Zhai, Qingya Wang, Li Ji, Hanyun Ren, Yujun Dong, Fan Yang, Yue Yin, Zeyin Liang, Qian Wang, Wei Liu, Yan Mei, Lu Zhang, Yuan Li

**Affiliations:** ^1^ Department of Hematology Peking University First Hospital Beijing China

**Keywords:** acute myeloid leukemia, core binding factor, hematopoietic stem cell transplantation, prognostic, risk stratifocation strategy

## Abstract

**Background:**

Chromosomal translocations involving core binding factor (CBF) genes account for 15% of adult acute myeloid leukemia (AML) cases in China. Despite being classified as favorable‐risk by European Leukemia Net (ELN), CBF‐AML patients have a 40% relapse rate. This study aims to analyze clinical characteristics and prognosis of CBF‐AML, compare its subtypes (inv(16) and t(8;21)), and validate prognostic factors.

**Methods:**

Retrospective analysis of 149 AML patients (75 CBF‐AML, 74 non‐CBF) at Peking University First Hospital (March 2012–March 2022).

**Results:**

CBF‐AML patients have significantly lower disease‐free survival (DFS) (*p* = 0.005) and higher non‐relapse mortality (NRM) (*p* = 0.028) compared to non‐CBF AML. inv (16) and t(8;21) show distinct co‐occurring gene mutation patterns, with inv(16) being prone to central nervous system (CNS) leukemia. Multivariate analysis identifies age as a risk factor for overall survival (OS) and disease free survival (DFS), kinase mutation as a risk factor for DFS and Recurrence, while WT1 mutation as a risk factor for OS and non relapse mortality (NRM) risk in t(8;21) AML. Allogeneic hematopoietic stem cell transplantation (allo‐HSCT) improves prognosis in low‐risk t(8;21).

**Conclusion:**

Prognosis of CBF‐AML is poorer than ELN guidelines suggest. inv(16) and (8;21) are separate entities with relatively poor prognoses, requiring rational risk stratification strategies. Allo‐HSCT may benefit low‐risk t(8;21), but further research is needed for conclusive evidence.

## Graphical abstract

According to the 2022 risk stratification guidelines by the European Leukemia Net (ELN), core binding factor (CBF) AML is classified as a favorable‐risk group. However, the relapse rate in CBF‐AML patients can reach up to 40%. This study indicated that the prognosis of CBF‐AML is poorer than ELN guidelines suggest. CBFβ‐AML and ETO‐AML are two separate entities with relatively poor prognoses, highlighting the need for the development of more rational risk stratification strategies. Allogeneic hematopoietic stem cell transplantation may benefit low‐risk ETO‐AML, although further research is warranted for conclusive evidence.

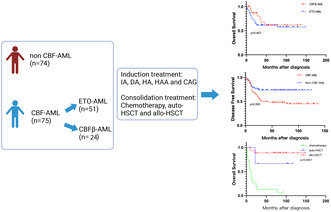

## 1 INTRODUCTION

As the most common subtype of acute myeloid leukemia (AML) with cytogenetic abnormalities, core binding factor (CBF) AML accounts for approximately 25% of pediatric AML patients and 15% of adults. It can achieve a higher complete remission (CR) rate and long‐term survival rate of 50%–60% after standardized induction therapy.^1^, ^2^, ^3^ In the 2022 European Leukemia Net (ELN) risk stratification, it is classified as a favorable‐risk group. However, through long‐term follow‐up of a large number of CBF‐AML patients, it has been found that the relapse rate in this group can be as high as 40%, with a median overall survival (OS) of less than 5 years, indicating clinical and genetic heterogeneity within this subtype.^4^, ^5^, ^6^, ^7^

Currently, there is sufficient evidence to suggest that the sole presence of abnormal CBF fusion proteins does not cause leukemia. Leukemic precursor cells harboring the RUNX1‐RUNX1T1 or CBFB‐MYH11 fusion genes have been shown to require at least 10 years to progress to clinical leukemia. This process involves the cooperative action of other gene mutations. Therefore, CBF‐AML is considered a multi‐step disease mechanism model.^2^, ^7^, ^8^, ^9^ Activating gene mutations in tyrosine kinase signaling, such as KIT, N/KRAS, and FLT3, are common in both subtypes.^10^ Approximately 20%–45% of CBF‐AML patients have KIT mutations,^11^ which are associated with a higher risk of relapse.^12^ In addition to activating gene mutations in tyrosine kinase signaling, there are also other gene mutations present in CBF‐AML, such as epigenetic regulatory gene mutations and cohesion complex gene mutations.^2^ The presence of these mutated genes may be associated with the prognosis of CBF‐AML.^8^ Therefore, further analysis of the clinical characteristics and genetic abnormalities of CBF‐AML is needed to improve treatment and prognosis.

Chromosomal rearrangements are the underlying mechanisms of CBF‐AML. The translocation event t(8;21)(q22;q22.1) and inversion inv(16)(p13.1;q22) disrupt the normal deoxyribonucleic acid (DNA) binding of heterodimers, resulting in the production of abnormal fusion genes, namely AML1‐ETO and CBFβ‐MYH11, respectively. These fusion genes interrupt normal transcription programs and cause a halt in the maturation of hematopoietic stem cells.^2^, ^13^ Due to the similar pathogenesis of CBF transcription factor abnormalities, t(8;21) and inv(16) are often reported together in clinical studies.^8^ However, previous clinical research has suggested differences in biological and clinical characteristics between these two subtypes. Therefore, it is necessary to conduct statistical analysis for these two distinct subtypes of CBF‐AML.

This study aims to analyze the case data of patients with CBF‐AML and non‐CBF AML who have been treated at Peking University First Hospital since 2012. The analysis will primarily focus on examining the clinical characteristics, prognosis, and risk factors associated with CBF‐AML. It aims to explore the prognostic factors and their impact on CBF‐AML outcomes. Furthermore, the study will compare the distinctions between inv(16) and t(8;21), aiming to uncover more rational methods for risk stratification and treatment strategies for CBF‐AML.

## 2 PATIENTS AND METHODS

### 2.1 Patients

This study is a retrospective cohort investigation that aimed to examine a group of patients diagnosed or treated for CBF‐AML at Peking University First Hospital between March 2012 and March 2022. The inclusion criteria were patients aged 14 years or older and diagnosed AML according to the 2016 World Health Organization classification for hematopoietic and lymphoid tumors.^14^ The CBF‐AML group consisted of 75 individuals, including 24 inv(16) patients and 51 t(8;21) patients. To establish a comparable control group for the same period, we employed the 2022 ELN risk classification, incorporating genetic factors at initial diagnosis, consolidation therapy, age, and sex. The matching process followed a 1:1 ratio, with predetermined criteria ensuring equivalence (identical categorical variables and specified ranges for continuous variables). In cases of multiple matches, a random selection was made. Ultimately, we successfully enrolled 74 non‐CBF patients as the matching control group.^15^

### 2.2 Detection

Cooperative mutation gene detection methods include next‐generation sequencing (NGS) and real‐time polymerase chain reaction (RT‐PCR). Cytogenetic abnormality detection methods include chromosomal karyotyping analysis and Fluorescence in Situ Hybridization (FISH).

### 2.3 Treatment plan

According to the Chinese Adult AML (Non‐Acute Promyelocytic Leukemia) Diagnosis and Treatment Guidelines, several induction treatment protocols are recommended, including IA (Idarubicin plus Cytarabine), DA (Daunorubicin plus Cytarabine), VA (Venetoclax plus Cytarabine), MA (Mitoxantrone plus Cytarabine), HA (Homoharringtonine plus Cytarabine), HAE (Homoharringtonine plus Cytarabine and Etoposide), HAA(Homoharringtonine plus Cytarabine and Aclacinomycin), D‐HAA (Decitabine plus HAA), AZA‐HAG (Azacitidine plus Homoharringtonine, Cytarabine and G‐CSF), CAG (Cytarabine plus Aclacinomycin and G‐CSF), and DCAG (Decitabine plus CAG). The choice of induction therapy is made after thorough discussion between the medical team and the patients, taking into consideration factors such as patient's economic status and drug tolerability. Consolidation treatment options encompass high‐dose cytarabine, priming chemotherapy, allogeneic hematopoietic stem cell transplantation, and autologous hematopoietic stem cell transplantation.^16^ For patients displaying neurological symptoms without intracranial hemorrhage or mass detected by CT or MR, lumbar puncture (LP) is warranted. If leukemia cells are present in the cerebrospinal fluid, administer intrathecal injection of Ara‐C (40–50 mg per session) and/or methotrexate (MTX, 5–15 mg per session) + dexamethasone (5–10 mg per session) concurrently with systemic chemotherapy. Intrathecal chemotherapy should be administered twice weekly until cerebrospinal fluid normalization, followed by once a week for 4–6 weeks. Patients with intracranial/spinal masses or elevated intracranial pressure are advised to undergo radiotherapy initially. Subsequently, intrathecal chemotherapy should be administered twice a week until cerebrospinal fluid normalization, followed by once a week for 4–6 weeks.

### 2.4 Treatment response assessment and definitions

CR, all of the following criteria should be met and maintained for >4 weeks: bone marrow blasts <5%, no evidence of extramedullary disease, neutrophil count >1.0 × 10^9^/L, platelet count >100 × 10^9^/L. Relapse, reappearance of leukemic blasts in peripheral blood, bone marrow blasts exceeding 5%, or extramedullary relapse. Overall survival (OS) is defined as the time from diagnosis until death or loss to follow‐up. Disease‐free survival (DFS) is defined as the time from diagnosis until induction failure, relapse, death, or loss to follow‐up. Relapse rate (RR) is defined as the time from diagnosis until induction failure or relapse. Non‐relapse mortality (NRM) is defined as the time from diagnosis until death during continuous remission.

### 2.5 Statistical analyses

Mann–Whitney *U*‐test was used to assess categorical variables, while the standard χ2 test was employed to evaluate continuous variables. Kaplan–Meier method was utilized to estimate OS, DFS, NRM, and RR, with log‐rank test employed for univariate comparisons. Variables with *p* values less than 0.15 in univariate analysis were included in multivariate analysis. Multivariate analysis was conducted using Cox regression analysis model. All statistical analyses were performed using the Statistical Package for the Social Sciences (SPSS) version 26.0 (SPSS Inc, IBM Corp, Armonk, NY, USA). A *p* < 0.05 was considered statistically significant. All event times were calculated from the date of diagnosis.

## 3 RESULTS

### 3.1 Clinical characteristics

In this single‐center retrospective analysis, we investigated the clinical characteristics of 75 patients with CBF‐AML and 74 patients with non CBF‐AML. To ensure a robust comparison, the CBF‐AML and non CBF‐AML groups were carefully matched based on ELN risk stratification. We conducted a comparative analysis of clinical features between CBF AML and non‐CBF AML. Variances in cytogenetic categories were observed, with CBF‐AML showing a higher propensity for Sex chromosome abnormalities (*p* = 0.031) and Complex karyotype (*p* = 0.001). Additionally, a significant distinction in platelet count at initial diagnosis was noted between CBF‐AML and non‐CBF‐AML (*p* = 0.018). (Table 1).

**TABLE 1:** Clinical characteristics of core binding factor acute myeloid leukemia and non core binding factor myeloid leukemia.

	Non‐CBF‐AML (*n* = 74)	CBF‐AML (*n* = 75)	*p*
ELN risk classification			
Favorable	50 (67.5%)	57 (76%)	0.121
Intermediate	17 (22.9%)	8 (10.6%)	
High	7 (9.4%)	10 (13.3%)	
Sex			
Male	47 (63.5%)	47 (62.6%)	0.915
Female	27 (36.4%)	28 (37.3%)	
Age (years)	44 (16–75)	38 (14–77)	0.162
Specific mutations			
CEBPA	27 (36.4%)	1 (1.3%)	
NPM1	33 (44.5%)	2 (2.6%)	
FLT3	14 (18.9%)	10 (13.3%)	
TP53	1 (1.3%)	1 (1.3%)	
IDH	5 (6.7%)	2 (2.6%)	
Induction therapy			
IA	39 (52.7%)	30 (40%)	0.120
Others	35 (47.2%)	45 (60%)	
Consolidation therapy			
Chemotherapy	16 (21.6%)	27 (36%)	0.072
Auto‐HSCT	5 (6.7%)	10 (13.3%)	
Allo‐HSCT	52 (70.2%)	37 (49.3%)	
UCBT	1 (1.3%)	1 (1.3%)	
ECOG	1 (0–4)	1 (0–4)	0.270
Type of AML			0.067
De novo	65 (87.8%)	72 (96%)	
Secondary	9 (12.1%)	3 (4%)	
Cytogenetic categories			
Autosomal abnormalities	5 (6.7%)	12 (16%)	0.129
Sex chromosome abnormalities	3 (4.1%)	11 (14.7%)	0.031
Complex karyotype	0	10 (13.3%)	0.001
WBC (×10^9^/L)	12.29 (0.67–311.28)	12.79 (0.95–391.60)	0.522
HGB (g/L)	88.5 (35–158)	80 (35–139)	0.086
PLT (×10^9^/L)	38 (5–430)	25 (5–424)	0.018
BM blasts (%)	61.5 (14–99)	52.5 (20–96.5)	0.434
Extramedullary involvement	3 (4%)	1 (1.3%)	0.603
CNS leukemia	4 (5.4%)	8 (10.6%)	0.370

Abbreviations: Allo‐HSCT, allogeneic stem cell transplantation; auto‐HSCT, autologous stem cell transplantation; BM, bone marrow; CNS, central nervous system; ELN, European Leukemia Net; HGB, hemoglobin; Mut, mutation; Others induction therapy contains DA, VA, MA, HA, HAE, HAA, CAG, DCAG, AZA‐HAG, Priming; PLT, palatelete; UCBT, umbilical cord blood transplantation; WBC, white blood cell.

CBF‐AML group comprising 24 inv(16) patients and 51 t(8;21) patients (Table 2). We initially conducted a thorough comparative analysis of the clinical features associated with inv(16) and t(8;21) chromosomal aberrations. Our findings illuminated distinct differences in various clinical characteristics, with notable variations between the two groups. Specifically, patients with inv(16) manifested a significantly higher frequency of Spliceosome mutations (*p* = 0.037) and WT1 mutations (*p* = 0.039) compared to their t(8;21) counterparts. Moreover, discernible variances in hematologic parameters were identified. Inv(16) patients exhibited elevated peripheral blood white blood cell counts at the time of diagnosis (*p* = 0.001) and higher bone marrow blast percentages at diagnosis (*p* = 0.002). In contrast, patients with t(8;21) demonstrated lower levels of peripheral blood hemoglobin at the point of diagnosis (*p* = 0.002). These findings not only underscore the clinical heterogeneity between inv(16) and t(8;21) subtypes but also contribute valuable insights into the distinctive molecular and hematologic profiles associated with each chromosomal aberration in the context of the studied population.

**TABLE 2:** Clinical characteristics of core binding factor acute myeloid leukemia.

	Inv (16) (*n* = 24)	T(8;21) (*n* = 51)	*p*
ELN risk classification			
Favorable	17 (70.8%)	41 (80.3%)	0.325
Intermediate	4 (16.6%)	3 (5.8%)	
Adverse	3 (12.5%)	7 (13.7%)	
Sex (male)	15 (62.5%)	32 (62.7%)	0.984
Age	41 (17–74)	38 (4–77)	0.218
Activating kinase mutation	11 (45.8%)	18 (35.2%)	0.382
Chromatin modifier	1 (4.1%)	2 (3.9%)	0.960
Transcription factor	0	2 (3.9%)	0.325
Cohesin	0	0	/
DNA methylation	5 (20.8%)	5 (9.8%)	0.190
Tumor suppressor	16 (66.6%)	22 (43.1%)	0.057
Spliceosome	2 (8.3%)	0	0.037
Kit_mut_	8 (33.3%)	9 (17.6%)	0.130
Flt3_mut_	5 (20.8%)	5 (9.8%)	0.492
WT1_mut_	16 (66.6%)	21 (41.5%)	0.039
Non‐mut	5 (21%)	16 (31.3%)	0.343
Cytogenetic categories			
Autosomal abnormalities	3 (12.5%)	9 (17.6%)	0.571
Sex chromosome abnormalities	1 (4.1%)	10 (19.6%)	0.078
Complex karyotype	1 (4.1%)	9 (17.6%)	0.109
Extramedullary involvement	0	1 (1.9%)	0.490
CNS leukemia	5 (20.8%)	3 (5.8%)	0.051
WBC(×10^9^/L)	28.24 (4.6–391.6)	9.8 (0.95–124.99)	0.001
HGB(g/L)	89 (55–139)	70.5 (35–132)	0.002
PLT(×10^9^/L)	36 (9–424)	20.9 (5–184)	0.06
BM blasts (%)	66.75 (20–96.5)	46.5 (20–94.5)	0.002
ECOG	1 (1–2)	1 (−4)	0.416
Type of AML			
De novo	22 (91.6)	50 (98%)	0.189
Secondary	2 (8.3%)	1 (1.9%)	
Induction chemotherapy			
IA	10 (41.6%)	20 (39.2%)	0.840
Others	14 (58.3%)	31 (60.7%)	
Consolidation therapy			
Chemotherapy	11	16	0.538
Auto‐HSCT	2	8	
Allo‐HSCT	11	26	
UCBT	0	1	

Abbreviations: Allo‐HSCT, allogeneic hematopoietic stem cell transplantation; BM, bone marrow; CNS, central nervous system; HGB, hemoglobin; Mut, mutation; Others induction therapy contains DA, VA, MA, HA, HAE, HAA, CAG, DCAG, AZA‐HAG, Priming; PLT, palatelete; WBC, white blood cell.

### 3.2 Outcomes analysis

**FIGURE 1:**
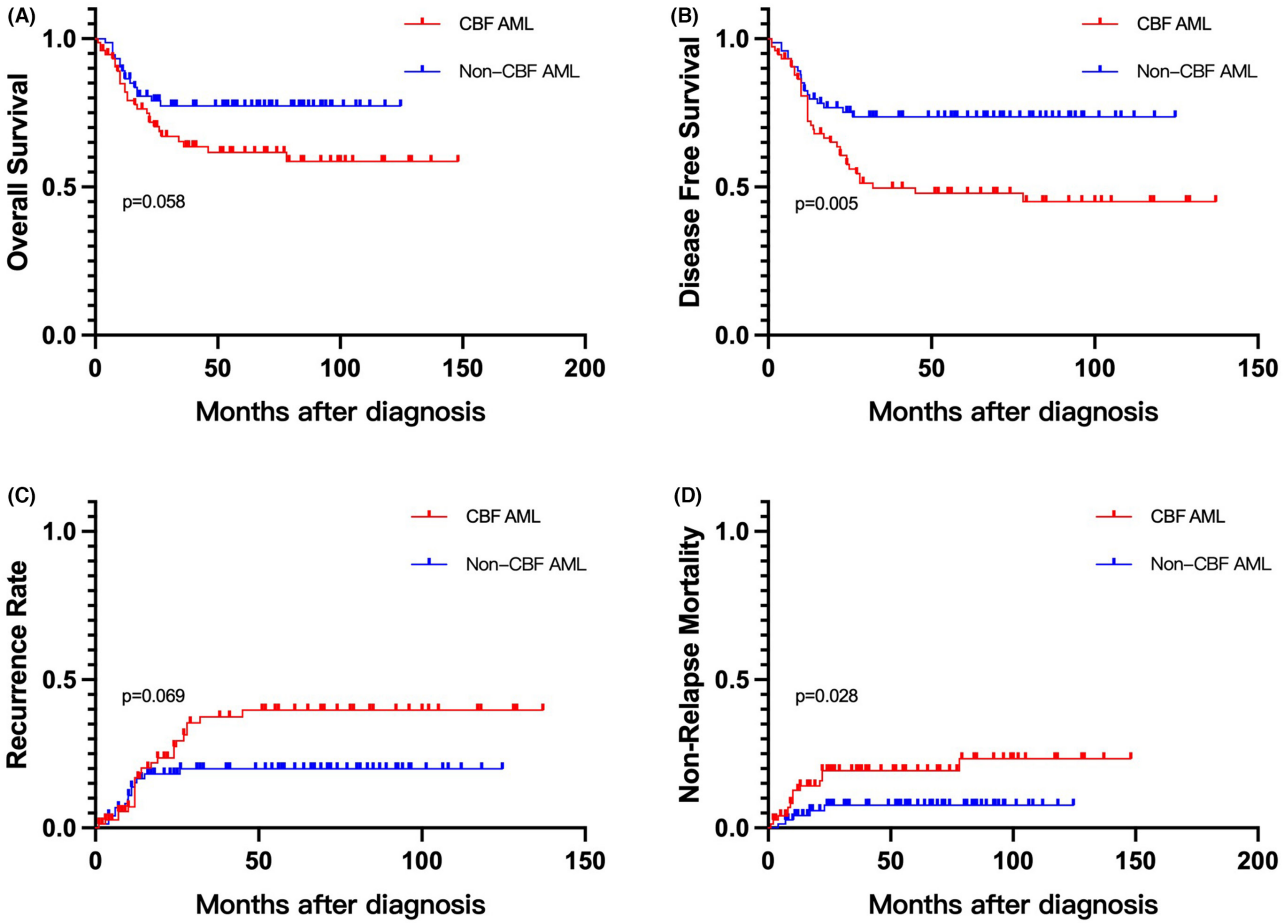
(A) Overall survival, (B) disease‐free survival, (C) recurrence rate, and (D) non‐relapse mortality for non‐CBF AML and CBF‐AML.

For the entire CBF‐AML cohort, the 3‐year OS, RR, DFS and NRM of CBF‐AML were 63.6%, 37.5%, 49.6%, and 19.3%, respectively. The 3‐year OS, DFS, RR and NRM for the non CBF‐AML control group were 77.3%, 73.6%, 19.9% and 7.7%, respectively. Compared to the non CBF‐AML group, CBF‐AML had significantly reduced DFS and increased NRM, *p* values are 0.005 and 0.028, respectively. (Figure 1).

**FIGURE 2:**
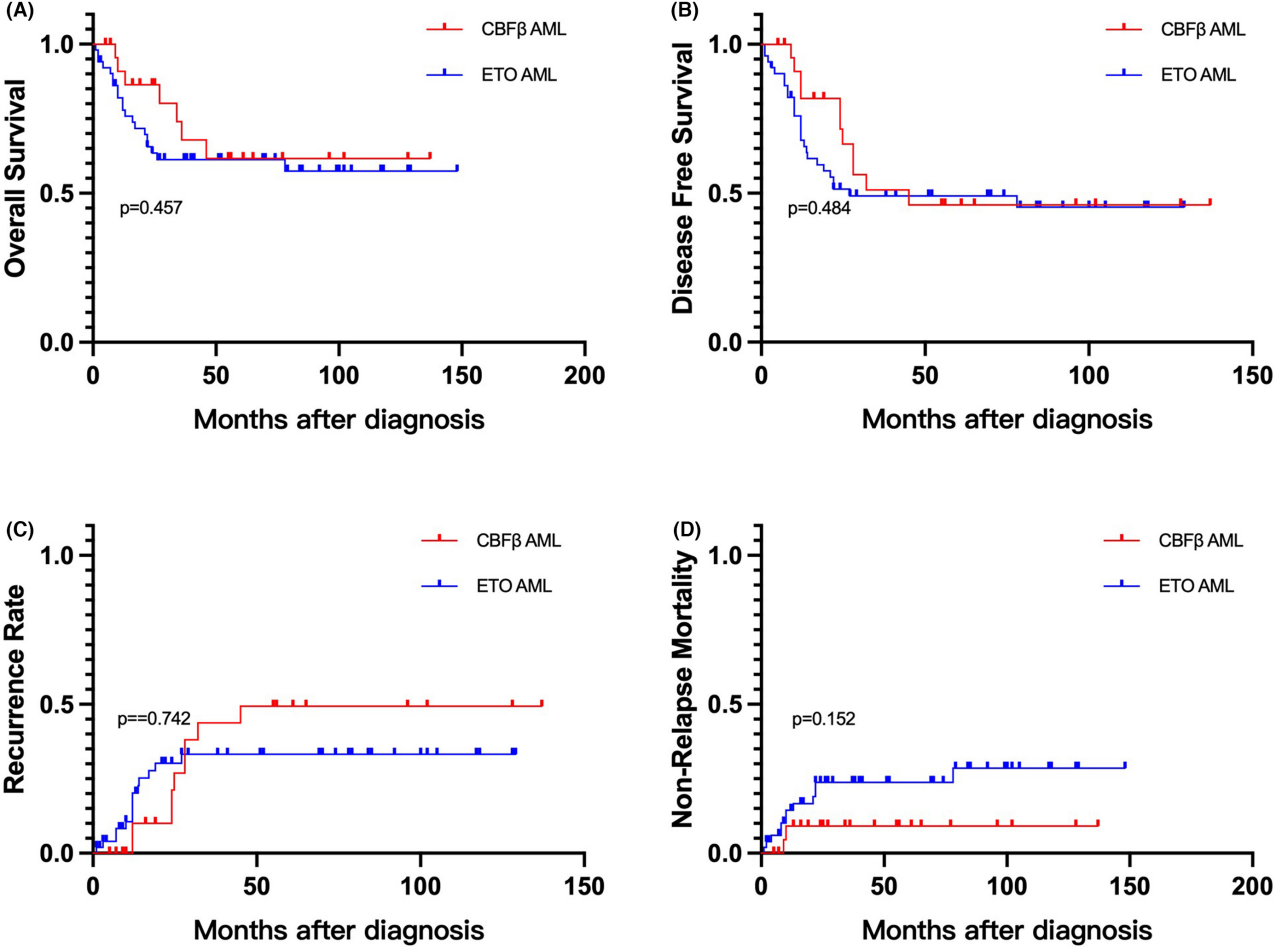
(A) Overall survival, (B) disease free survival, (C) recurrence rate, and (D) non‐relapse mortality for ETO‐ AML and CBFβ‐AML.

For t(8;21), the 3‐year OS, RR, DFS, and NRM were 61.3%, 33.2%, 49.1%, and 23.8%, respectively. inv(16) demonstrated a 3‐year OS, RR, DFS, and NRM of 67.9%, 43.8%, 51.1%, and 9.1%, respectively. Compared to inv(16), t(8;21) did not show significant differences in OS, RR, DFS, and NRM. (Figure 2).

### 3.3 Prognostic factors analysis

We subsequently assessed the risk factors associated with OS, DFS, NRM, and RR. In the inv(16) subgroup, noteworthy associations emerged: patients undergoing IA induction chemotherapy exhibited a lower Overall OS (*p* = 0.043), while secondary AML cases demonstrated an increased susceptibility to higher NRM (*p* = 0.016). Moreover, individuals diagnosed with lower hemoglobin (HGB) levels exhibited an elevated RR (*p* = 0.037). Additionally, within the inv(16) cohort presenting concomitant autosomal abnormalities, a trend toward higher NRM was observed (*p* = 0.097), although this distinction did not achieve statistical significance. In the subsequent multivariate analysis, the protective role of higher HGB levels against RR was affirmed, with hazard ratios (HRs) of 0.963 (95% confidence interval [CI]: 0.934–0.994) (Table 3).

**TABLE 3:** Univariate and multivariate analyses in OS, DFS, RR and NRM for inv (16)‐AML.

	OS		DFS		RR		NRM	
	Univariate analysis	Multivariate analysis	Univariate analysis	Multivariate analysis	Univariate analysis	Multivariate analysis	Univariate analysis	Multivariate analysis
Age	0.898		0.785		0.879		0.326	
Sex	0.330		0.799		0.404		0.276	
Activating kinase	0.456		0.276		0.267		0.816	
Chromatin modifier	0.479		0.365		0.395		0.755	
Transcription factor	NA		NA		NA		NA	
Cohesin	NA		NA		NA		NA	
DNA methylation	0.763		0.293		0.487		0.371	
Tumor suppressor	0.609		0.356		0.614		0.276	
Spliceosome	0.414		0.305		0.358		0.651	
Kit_mut_	0.882		0.166		0.198		0.599	
Flt3_mut_	0.761		0.902		0.852		0.569	
WT1_mut_	0.609		0.356		0.614		0.276	
Non‐mutation	0.370		0.127	0.168	0.185		0.436	
Autosomal abnormalities	0.477		0.792		0.395		0.097	0.950
Sex chromosome abnormalities	0.176		0.627		0.507		0.755	
Complex karyotype	NA		NA		NA		NA	
WBC	NA		NA		NA		NA	
HGB	0.925		0.300		0.037	0.018	0.243	
PLT	0.321		0.327		0.517		0.363	
BM blasts	0.955		0.594		0.771		0.499	
Extramedullary involvement	NA		NA		NA		NA	
CNS leukemia	0.488		0.987		0.726		0.499	
ELN risk classification	0.367		0.439		0.134	0.266	0.738	
ECOG PS	NA		NA		NA		NA	
Type of AML	0.477		0.729		0.395		0.016	0.950
Induction therapy	0.043	0.487	0.168		0.463		0.112	0.908
Consolidation therapy	0.367		0.411		0.453		0.886	

Abbreviations: BM, bone marrow; CNS, central nervous system; DFS, disease free survival; HGB, hemoglobin; Mut, mutation; NRM, non‐relapse mortality; OS, overall survival; PLT, platelets; RR, recurrence rate; WBC, white blood cell.

In the t(8;21) group, increasing age was associated with higher RR (*p* = 0.008), lower OS (*p* = 0.000), lower DFS (*p* = 0.000), and higher NRM (*p* = 0.009). Patients with concomitant tyrosine kinase gene mutations had a higher RR (*p* = 0.021) and lower DFS (*p* = 0.023). Patients with combined KIT mutations had lower OS (*p* = 0.048). Patients with combined WT1 mutations had a higher NRM (*p* = 0.046). Patients with coexisting sex chromosome abnormalities had lower DFS (*p* = 0.05). Moreover, a higher proportion of bone marrow blasts at initial diagnosis was associated with lower OS (*p* = 0.029) and higher NRM (*p* = 0.071) in these patients. Higher ECOG PS is associated with lower OS (*p* = 0.016). IA induction chemotherapy is associated with higher DFS (*p* = 0.003), lower NRM (*p* = 0.025). The results of the multivariate analysis revealed that age, KIT mutation and WT1 mutation were confirmed as risk factors for OS, with hazard ratios (HRs) of 1.047 (95% CI: 1.002–1.095), 8.676 (95% CI: 1.830–41.141) and 4.021 (95% CI: 1.377–11.742), respectively. Additionally, age, activating kinase mutation and induction chemotherapy were identified as risk factors for disease‐free survival (DFS) with HRs of 1.036 (95% CI: 1.002–1.071), 8.236 (95% CI: 2.713–25.001) and 7.437 (95% CI: 1.968–28.102), respectively. Activating kinase and induction chemotherapy were found to be risk factors for relapse rate (RR) with HRs of 7.903 (95% CI: 1.528–40.883) and 6.055 (95% CI: 1.331–27.540), respectively. Moreover, WT1 mutation was determined to be a risk factor for non‐relapse mortality (NRM) with HRs of 11.521 (95% CI: 18.85–70,481). Results are summarized in Table 4.

**TABLE 4:** Univariate and multivariate analysis in OS, DFS, RR, and NRM for t(8;21)‐AML.

	OS		DFS		RR		NRM	
	Univariate analysis	Multivariate analysis	Univariate analysis	Multivariate analysis	Univariate analysis	Multivariate analysis	Univariate analysis	Multivariate analysis
Age	0.000	0.040	0.000	0.037	0.008	0.239	0.009	0.379
Sex	0.396		0.594		0.392		0.891	
Activating kinase	0.163		0.023	0.000	0.021	0.014	0.596	
Chromatin modifier	0.644		0.927		0.458		0.357	
Transcription factor	0.311		0.900		0.642		0.450	
Cohesin	NA		NA		NA		NA	
DNA methylation	0.998		0.845		0.507		0.264	
Tumor suppressor	0.165		0.393		0.624		0.066	
Spliceosome	NA		NA		NA		NA	
Kit_mut_	0.048	0.007	0.208		0.204		0.545	
Flt3_mut_	0.518		0.430		0.057	0.869	0.286	
WT1_mut_	0.108	0.011	0.260		0.763		0.046	0.008
Autosomal abnormalities	0.696		0.745		0.701		0.362	
Sex chromosome abnormalities	0.169		0.05	0.360	0.368		0.063	0.970
Complex karyotype	0.239		0.620		0.832		0.285	
WBC	0.410		0.615		0.373		0.364	
HGB	0.964		0.267		0.283		0.682	
PLT	0.297		0.845		0.344		0.364	
BM blasts	0.029	0.251	0.06	0.421	0.684		0.071	0.920
Extramedullary involvement	0.480		0.392		0.519		0.598	
CNS leukemia	0.702		0.904		0.953		0.880	
ELN	0.367		0.671		0.995		0.478	
ECOG PS	0.016	0.128	0.059	0.298	0.495		0.035	0.460
Type of AML	0.452		0.702		0.319		0.557	
Induction therapy	0.057	0.202	0.003	0.003	0.083	0.020	0.025	0.091
Consolidation therapy	0.000	0.536	0.000	0.801	0.025	0.992	0.014	0.888

Abbreviations: BM, bone marrow; CNS, central nervous system; DFS, disease free survival; HGB, hemoglobin; Mut, mutation; NRM, non‐relapse mortality; OS, overall survival; PLT, platelets; RR, recurrence rate; WBC, white blood cell.

In the non CBF‐AML group, Increased age, higher ECOG, non‐IA induction therapy and chemotherapy consolidation therapy were associated with lower OS (*p* = 0.014, *p* = 0.003, *p* = 0.019 and *p* = 0.000, respectively). Increased age, higher ECOG and chemotherapy consolidation therapy were associated with lower DFS (*p* = 0.029, *p* = 0.023 and *p* = 0.003, respectively). Increased age was associated with higher RR (*p* = 0.029). Multivariate analysis confirmed that extramedullary involvement was a risk factor for NRM with an HR of 20.058 (95% CI: 1.042–386.294) (*p* = 0.047). (Table S1).

### 3.4 Subgroup analysis

We divided inv(16) and t(8;21) into favorable and intermediate/high‐risk subgroups according to ELN stratification. We analyzed whether different consolidation treatment modalities (including chemotherapy, autologous transplantation, and allogeneic transplantation) had an impact on the prognosis of the different subgroups.

**FIGURE 3:**
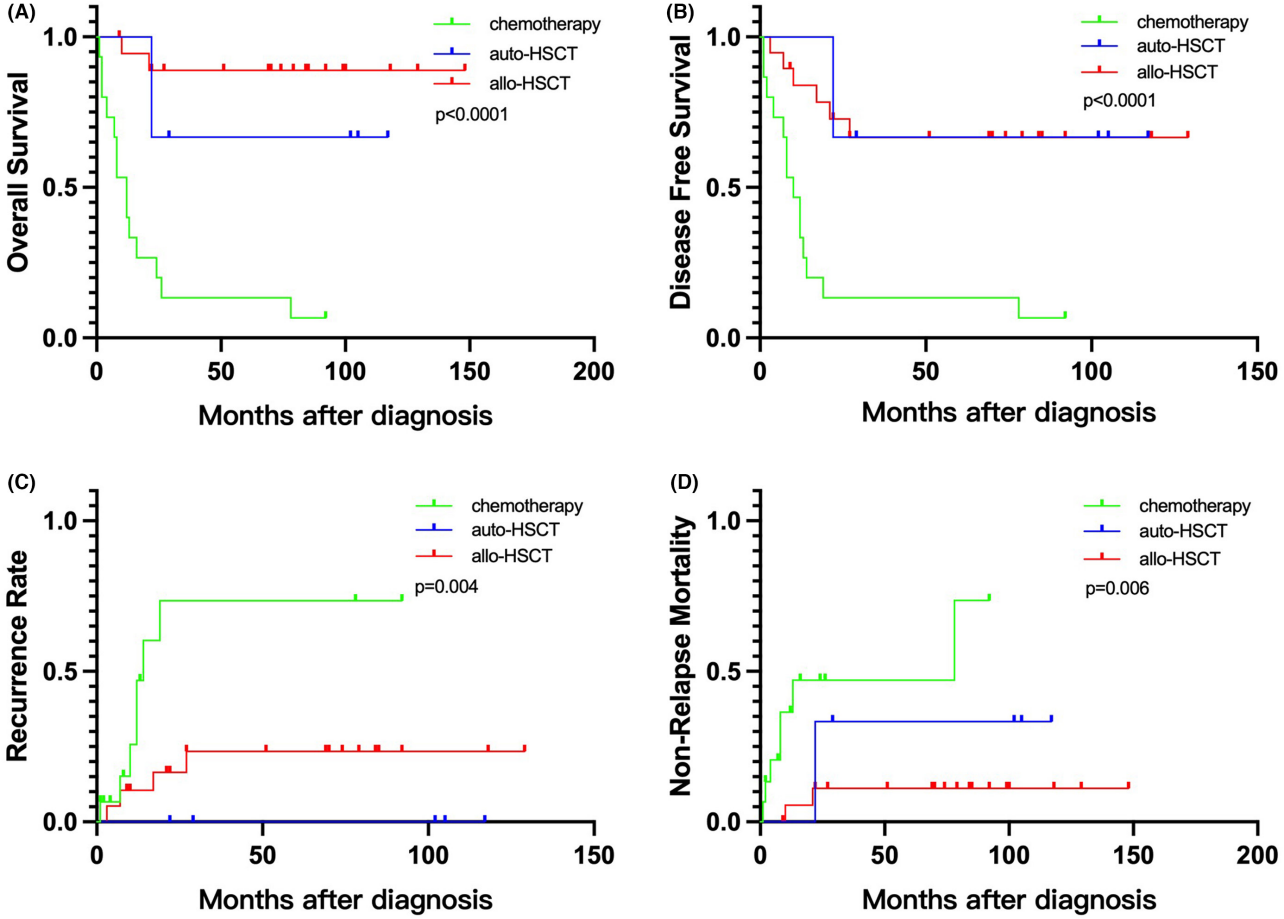
(A) Overall survival, (B) disease‐free survival, (C) recurrence rate, and (D) non‐relapse mortality for ELN favorable ETO‐AML according to different consolidation treatments.

**FIGURE 4:**
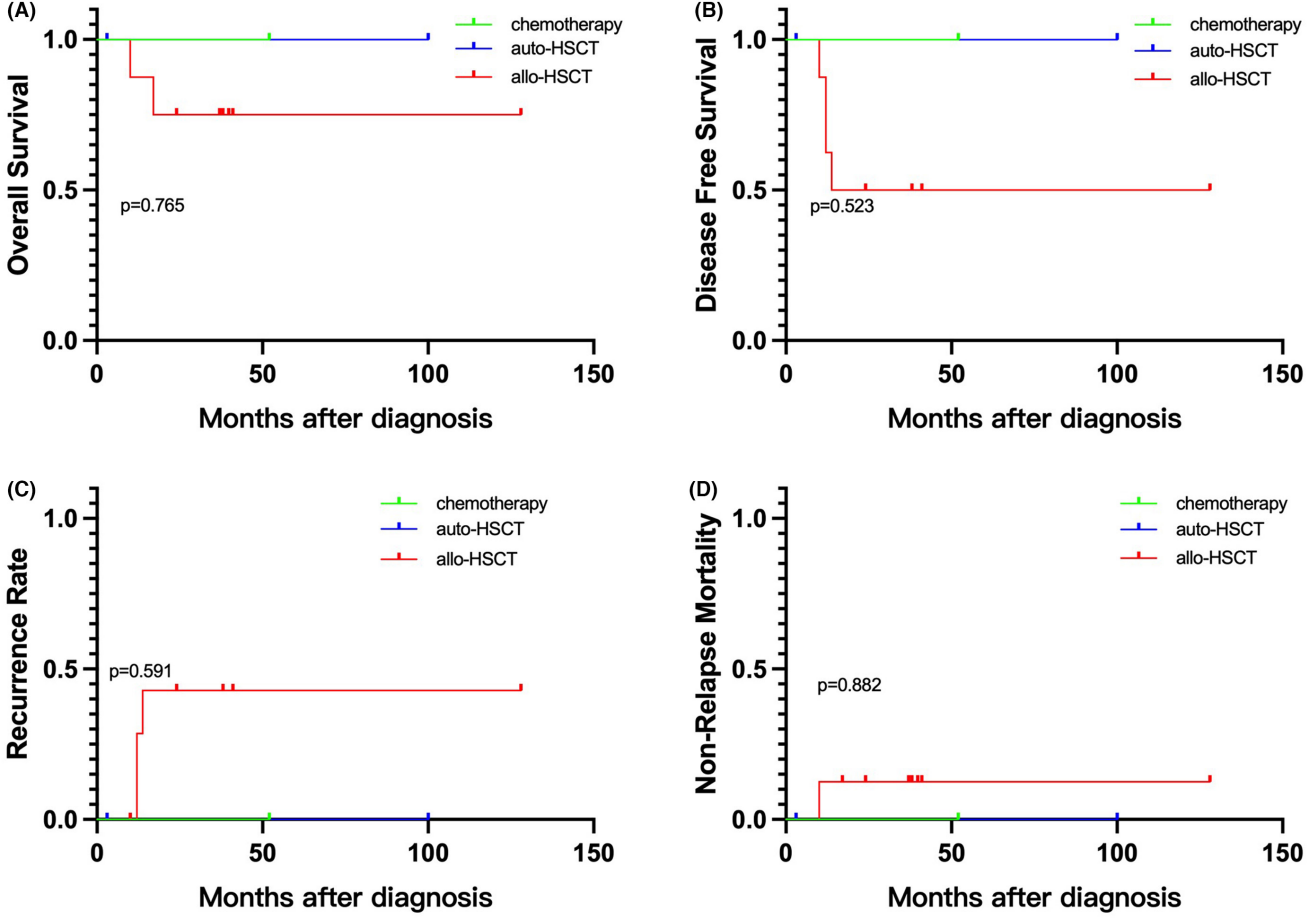
(A) Overall survival, (B) disease‐free survival, (C) recurrence rate, and (D) non‐relapse mortality for ELN intermediate/high‐risk ETO‐AML according to different consolidation treatments.

The results showed that in the favorable t(8;21) subgroup, which included 40 patients, there were 15 patients who received chemotherapy, 6 patients who underwent autologous hematopoietic stem cell transplantation (auto‐HSCT), and 19 patients who underwent allogeneic hematopoietic stem cell transplantation (allo‐HSCT). Compared to patients receiving chemotherapy, those who underwent auto‐HSCT had a lower RR (*p* = 0.010), higher OS (*p* = 0.007), and higher DFS (*p* = 0.003). Similarly, patients who underwent allo‐HSCT had a lower RR (*p* = 0.011), higher OS (*p* = 0.000), and higher DFS (*p* = 0.000) compared to those receiving chemotherapy. However, no significant difference in prognosis was observed between allo‐HSCT and auto‐HSCT transplantation patients. (Figure 3) In the intermediate/high‐risk t(8;21) subgroup, which included 11 patients, there was 1 patient who received chemotherapy, 2 patients who underwent autologous transplantation, and 8 patients who underwent allogeneic transplantation. Our study did not find any correlation between the choice of consolidation treatment modality and prognosis in this subgroup. (Figure 4).

In the favorable inv(16) subgroup, comprising six patients receiving chemotherapy, two patients undergoing auto‐HSCT, and nine patients undergoing allo‐HSCT, no significant correlation was observed between the choice of consolidation treatment modality and prognosis. Similarly, in the intermediate/high‐risk inv(16) subgroup, five patients received chemotherapy, no patients underwent auto‐HSCT, and two patients underwent allo‐HSCT. No significant association was found between the choice of consolidation treatment modality and prognosis in this subgroup as well.

## 4 DISCUSSION

This study analyzed the clinical characteristics of CBF‐AML and its two subtypes, inv(16) and t(8;21), in our center. First, consistent with previous literature, the most common co‐occurring gene mutations in CBF‐AML were kinase‐activating genes.^1^ Second, when comparing t(8;21) and inv(16), differences were observed in terms of clinical features, co‐occurring gene mutations, and cytogenetic abnormalities. Specifically, in terms of basic clinical features, inv(16) patients had higher peripheral blood leukocyte counts and higher bone marrow blast percentages at diagnosis, while t(8;21) patients had lower peripheral blood hemoglobin levels at diagnosis. In terms of co‐occurring gene mutations, Spliceosome and WT1 mutations were more commonly observed in inv(16). Furthermore, our inv(16) patients did not exhibit mutations in transcription factors or cohesion complex genes, which is consistent with previous studies.^12^, ^13^, ^14^, ^17^ These findings collectively suggest that inv(16) and t(8;21) are two distinct types of diseases and should not be simply categorized as the same disease. These results have important implications for treatment strategies and improving prognosis.

In order to explore the prognostic factors of inv(16) and t(8;21), we conducted univariate and multivariate analyses. Univariate analysis revealed that the presence of co‐occurring kinase gene mutations was associated with poor prognosis in t(8;21). Kinase gene mutations were associated with a higher relapse rate and lower disease free survival, while WT1 mutations were associated with a higher non‐relapse mortality rate. Studies have shown that WT1 is often regarded as an oncogene in leukemia.^18^ Previous studies have suggested that WT1 mutations may be associated with a decrease in OS and DFS.^19^, ^20^, ^21^ This also suggests that WT1 may serve as a risk factor affecting the prognosis of t(8;21), but further clinical data validation is needed in the future. Among our study subjects, KIT mutation and FLT3 mutation were the two most common types of kinase‐activating gene mutations. Further analysis indicated a correlation between KIT mutations and lower OS rates, which has also been reported in multiple previous studies.^11^, ^12^, ^22^, ^23^

According to the latest ELN risk stratification, similar to NPM1 mutations and CEBPA double mutations, inv(16) and t(8;21) are still classified into the favorable category.^15^ However, in our CBF‐AML group, the 3‐year OS was only 63.6%, compared to 77.3% in the control group. Despite matching the basic clinical characteristics between the control and CBF groups, our findings indicate that the CBF‐AML group exhibited a reduction in DFS and an increase in NRM when compared to non‐CBF‐AML patients. Consistent with previous studies, this suggests that t(8;21) and inv(16) may not simply be classified using the same risk stratification approach as non‐CBF‐AML, and new, more scientifically‐based risk stratification methods are needed.^19^

Currently, there is no consensus on the indications and timing of allo‐HSCT consolidation therapy for CBF‐AML. Some studies suggest that allo‐HSCT consolidation therapy can improve prognosis.^11^ There are also reports proposing that allo‐HSCT can effectively improve the prognosis of high‐risk t(8;21).^24^ Therefore, this study also analyzed the impact of consolidation therapy on prognosis. The data showed that in the favorable t(8;21) subgroup, individuals who received allo‐HSCT as consolidation therapy had longer OS and DFS. However, this phenomenon was not observed in intermediate/high‐risk t(8;21) patients. This result may be attributed to the inappropriate risk stratification, leading to the failure to timely identify relevant patients among intermediate to high‐risk CBF‐AML patients. It may also be related to the low sample size in the intermediate to high‐risk group.

The limitation of this study is its retrospective design, which can only indirectly reflect the clinical characteristics of CBF‐AML and the unresolved issues in the diagnostic and treatment processes. Future prospective studies with larger sample sizes are needed to provide further support.

## 5 CONCLUSION

In conclusion, this study found that in real world, inv(16) and t(8;21) are two distinct types of AML with relatively poorer prognosis compared to other low‐risk AML subtypes. Kinase mutations and WT1 may be associated with adverse outcomes for t(8;21). The ELN risk stratification alone cannot fully identify high‐risk patients, and alternative, more scientifically effective risk stratification methods are needed to accurately identify high‐risk CBF‐AML patients. Allo‐HSCT consolidation therapy may be effective in improving prognosis of some ELN favorable t(8;21).

## AUTHOR CONTRIBUTIONS

**Yamei Zhai:** Methodology (equal); writing – original draft (equal). **Qingya Wang:** Investigation (equal); methodology (equal); writing – original draft (equal). **Li Ji:** Methodology (equal). **Hanyun Ren:** Conceptualization (equal). **Yujun Dong:** Supervision (equal). **Fan Yang:** Methodology (equal). **Yue Yin:** Supervision (equal). **Zeyin Liang:** Supervision (equal). **Qian Wang:** Supervision (equal). **Wei Liu:** Supervision (equal). **Yan Mei:** Investigation (equal). **Lu Zhang:** Investigation (equal). **Yuan Li:** Conceptualization (equal); writing – review and editing (equal).

## FUNDING INFORMATION

This work was financially supported by National Natural Science Foundation of China (No.81970410); Beijing Natural Science Foundation (No.7202203); The Beijing Municipal Science and Technology Commission (No. Z221100007422008); Peking University First Hospital Scientific Research Seed Fund (No. 2021SF13).

## CONFLICT OF INTEREST STATEMENT

The authors declare that the research was conducted in the absence of any commercial or financial relationships that could be construed as a potential conflict of interest.

## ETHICS STATEMENT

The study was conducted in accordance with the Declaration of Helsinki and was approved by the institutional review board at Peking University First Hospital (No.2023‐332).

## PATIENT CONSENT FOR PUBLICATION

Due to the retrospective nature of the study, the requirement for written informed consent was waived. All data used in this manuscript were anonymized to ensure patient confidentiality.

## Supporting information


Table S1.

## Data Availability

The datasets generated during and/or analyzed during the current study are available from the corresponding author on reasonable request.
