# Supplementary material for: Clinical characteristics and prognostic factors analysis of core binding factor acute myeloid leukemia in real world
Source: Cancer Med. 2023 Dec 7;12(24):21592–604. doi: 10.1002/cam4.6693 (PMC10757144; doi:10.1002/cam4.6693)
Supplement: Supplementary file 1 — Table S1. [file CAM4-12-21592-s001.docx]

**Supplementary table 1.** Univariate and multivariate analysis in OS, DFS, RR and NRM for non CBF -AML

|  | OS | | DFS | | RR | | NRM | |
| --- | --- | --- | --- | --- | --- | --- | --- | --- |
|  | Univariate analysis | Multivariate analysis | Univariate analysis | Multivariate analysis | Univariate analysis | Multivariate analysis | Univariate analysis | Multivariate analysis |
| Age | 0.014 | 0.447 | 0.029 | 0.419 | 0.029 | 0.301 | 0.564 |  |
| Sex | 0.707 |  | 0.731 |  | 0.592 |  | 0.850 |  |
| WBC | 0.724 |  | 0.471 |  | 0.535 |  | 0.05 | 0.824 |
| PLT | 0.884 |  | 0.451 |  | 0.864 |  | 0.104 |  |
| HGB | 0.078 | 0.945 | 0.351 |  | 0.114 | 0.747 | 0.482 |  |
| BM blast cell percent | 0.543 |  | 0.238 |  | 0.768 |  | 0.082 |  |
| CEBPA | 0.324 |  | 0.340 |  | 0.516 |  | 0.432 |  |
| NPM1 | 0.274 |  | 0.825 |  | 0.882 |  | 0.437 |  |
| FLT3_mut_ | 0.942 |  | 0.733 |  | 0.258 |  | 0.206 |  |
| TP53_mut_ | 0.670 |  | 0.619 |  | 0.653 |  | 0.837 |  |
| IDH_mut_ | 0.279 |  | 0.222 |  | 0.288 |  | 0.558 |  |
| ECOG | 0.003 | 0.117 | 0.023 | 0.068 | 0.05 | 0.424 | 0.220 |  |
| Induction therapy | 0.019 | 0.076 | 0.161 |  | 0.068 | 0.228 | 0.799 |  |
| Consolidation therapy | 0.000 | 0.094 | 0.003 | 0.483 | 0.148 | 0.964 | 0.738 |  |
| Type of AML | 0.471 |  | 0.700 |  | 0.341 |  | 0.401 |  |
| Autosomal abnomalities | 0.936 |  | 0.797 |  | 0.951 |  | 0.540 |  |
| Sex cheomosome abnormalities | 0.633 |  | 0.793 |  | 0.553 |  | 0.648 |  |
| Complex karyotype | / |  | / |  | / |  | / |  |
| Extramedullary involvement | 0.026 | 0.835 | 0.084 | 0.639 | 0.453 |  | 0.024 | 0.047 |
| CNS leukemia | 0.295 |  | 0.958 |  | 0.792 |  | 0.561 |  |
| Activating kinase | 0.858 |  | 0.536 |  | 0.182 |  | 0.284 |  |
| WT1_mut_ | / |  | / |  | / |  | / |  |
| KIT_mut_ | 0.521 |  | 0.460 |  | 0.519 |  | 0.725 |  |
